# Supplementary material for: Identification and Characterization of Aptamers Targeting Ovarian Cancer Biomarker Human Epididymis Protein 4 for the Application in Urine
Source: Cancers (Basel). 2023 Jan 10;15(2):452. doi: 10.3390/cancers15020452 (PMC9856783; doi:10.3390/cancers15020452)
Supplement: Supplementary file 1 [file cancers-15-00452-s001.zip › cancers-2120359-supplementary.pdf]

cancers

Supplementary materials:

Identification and Characterization of Aptamers Targeting Ovarian Cancer Biomarker Human Epididymis Protein 4 for Application in Urine

Antonija Hanžek, Frédéric Ducongé, Christian Siatka and Anne-Cécile E. Duc

Table S1. Detailed Hi-Fi SELEX conditions for the selection of aptamers to ovarian cancer protein biomarker HE4. The aptamers are selected after 10 cycles of positive selection (+) to target 6xHis-HE4 and two rounds of counter selection (-) to 6xHis peptide in 1X urine with incubation for 1 hour at 25 °C. To obtain specific sequences, the selection stringency is increased by changing the amount of DNA and protein, while increasing the numbers, duration and buffers in washing process.

Cycle	DNA Source	DNA (nmol)	Protein (pmol)	Washing Steps	Washing Duration	Washing Buffers (400 µL)	n of ddPCR Cycles	n of PCR Cycles*
**C1+**	*Hi-Fi* library	1.25	200	1	2 min	W1 [1]	40	n/a
**C2+**	C1+	0.47	200	2	2 min	W1 (2 steps)		n/a
**C3+**	C2+	0.08	100	2	5 min	W1 (2 steps)		18
**C4+**	C3+	0.13	100	3	5 min	W1 (1 step)<br>W2 [2] (2 steps)		14
**C5+**	C4+	0.15	100	3	5 min	W1 (1 step)<br>W2 (2 steps)		14
**C6+**	C5+	0.04	200	3	5 min	W2 (3 steps)		18
**C7+**	C6+	0.03	200	3	5 min	W2 (3 steps)		10
**C8+**	C7 +	0.08	200	3	5 min	W2 (3 steps)		10
**C9+**	C8 +	0.10	200 HE4	3	10 min	W2 (3 steps)		15
**C9-**	C8+	0.10	200 6xHis peptide	3	10 min	W2 (3 steps)		15
**C10+**	C9+	0.10	100 HE4	3	10 min	W2 (3 steps)		8
**C10-**	C9-	0.10	100 6xHis peptide	3	10 min	W2 (3 steps)		8

* It is important to determine optimal number of PCR cycles in each SELEX cycle to obtain specific amplicon of expected size of 70 bp;
1 – Wash buffer 1 (WB1) – 1X urine; 2 – Wash buffer 2 (WB2) – 1X urine + 0.01 % Tween-20

Table S2. The detailed constitution and volumes of the PCR mixtures. The table provides details of the ddPCR and PCR reaction mixtures used in the selection of aptamers to ovarian cancer protein biomarker HE4.

	**Samples (Anti-HE4 Aptamers)**	**Negative PCR Control (Non Template Control, NTC)**	**Positive PCR Control (Initial Library)**
	**Digital droplet PCR –** ***partitioning (volume in µL)***		
**DNA template; µL**	**2** *(DNA aptamers recovered after selection to HE4, final concentration unknown)*	**2** *(initial DNA library, final < 10^{-7} ng DNA)*	**-** *(2 µL of water, no DNA)*
**Forward primer (from stock at 10 µM); µL**	**0.2** *(final concentration 100 nM)*	**0.2** *(final concentration 100 nM)*	**0.2** *(final concentration 100 nM)*
**Phosphorylated Reverse primer (from stock at 10 µM); µL**	**0.2** *(final concentration 100 nM)*	**0.2** *(final concentration 100 nM)*	**0.2** *(final concentration 100 nM)*
**PCR mix (EvaGreen ddPCR mix, 2X); µL**	**10** *(final concentration 1X)*	**10** *(final concentration 1X)*	**10** *(final concentration 1X)*
**Nuclease-free H_2O; µL**	**7.6**	**7.6**	**7.6**
**Total reaction volume; µL**	**20**	**20**	**20**
**Total number PCR reactions used in experiment/analysis**	**1**	**1**	**1**
	**Regular PCR –** ***reamplification to increase the yield (volume in µL)***		
**DNA template; µL**	**2** *(DNA aptamers extracted from ddPCR droplets,, final concentration unknown)*	**2** *(initial DNA library, final < 10^{-7} ng DNA)*	**-** *(2 µL of water, no DNA)*
**Forward primer (from stock at 10 µM); µL**	**4** *(final concentration 400 nM)*	**4** *(final concentration 400 nM)*	**4** *(final concentration 400 nM)*
**Phosphorylated Reverse primer (from stock at 10 µM); µL**	**4** *(final concentration 400 nM)*	**4** *(final concentration 400 nM)*	**4** *(final concentration 400 nM)*
**DreamTaq polymerase (5 U/µL); µL**	**0.5** *(final 2.5 U/ µL)*	**0.5** *(final 2.5 U/ µL)*	**0.5** *(final 2.5 U/ µL)*
**DreamTaq polymerase buffer supplied with 20 mM $MgCl_2$, 10X ; µL**	**10** *(final concentration 1X of buffer with 2 mM $MgCl_2$)*	**10** *(final concentration 1X of buffer with 2 mM $MgCl_2$)*	**10** *(final concentration 1X of buffer with 2 mM $MgCl_2$)*
**dNTPs (from stock at 10 µM each); µL**	**2** *(final concentration 0.2 mM each)*	**2** *(final concentration 0.2 mM each)*	**2** *(final concentration 0.2 mM each)*
**Nuclease-free H_2O; µL**	**77.5**	**77.5**	**77.5**
**Total reaction volume; µL**	**100**	**100**	**100**
**Total number PCR reactions used in experiment/analysis**	**30 (combined to a final volume of 3000 µL = 3 mL for continuting the SELEX)**	**1**	**1**

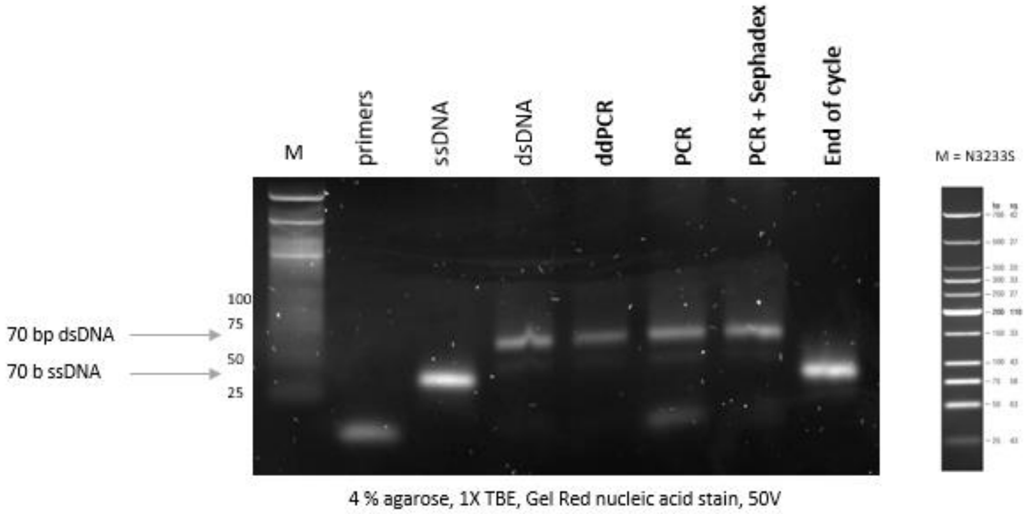

Figure S1. Gel electrophoresis of a typical cycle during Hi-Fi SELEX to ovarian cancer biomarker HE4. The figure represents electrophoresis gel (4 % agarose, 1X TAE, GelRed nucleic acid staining, 50V) of the each validation step during one Hi-Fi selection cycle to protein HE4 (here, as example: cycle 8). It is important to confirm proper amplification after ddPCR/PCR and obtain specific anti-HE4 aptamer amplicon of expected size (70 bp) ideally without by-products, as well as ensure primer removal by Sephadex G-50. At the end of cycle, it is important to confirm proper ssDNA generation by lambda exonuclease with complete conversion of double stranded 70 bp DNA to single-stranded 70 bases DNA before using as input for next cycle. **M** = low molecular weight DNA ladder (ref. N3233S, New England Biolabs); **primers** = reference 20-bases DNA, primers; **ssDNA** = reference 70-bases DNA, original library; **dsDNA** = reference 70 bp DNA, amplified original library; **ddPCR** = dsDNA after ddPCR amplification and chloroform extraction; **PCR** = dsDNA after PCR re-amplification ; **PCR + Sephadex** = dsDNA after purification and primer removal with Sephadex G-50 columns; **End of cycle** = ssDNA, regenerated aptamer DNA after lambda exonuclease digestion (10 U/μL, 1h, 37 °C) at the end of selection cycle and input DNA used for the next cycle.

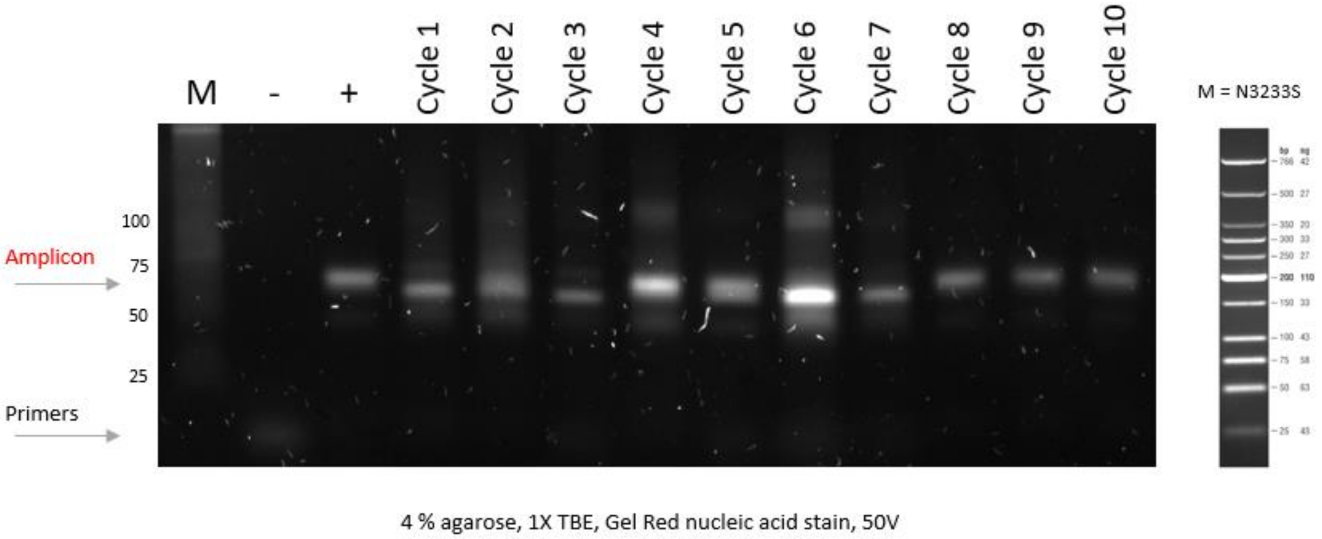

Figure S2. Gel electrophoresis of anti-HE4 aptamers during Hi-Fi SELEX to ovarian cancer biomarker HE4. The results show electrophoresis gel gel (4 % agarose, 1X TAE, GelRed nucleic acid staining, 50V) of the all cycles of Hi-Fi SELEX amplified DNA obtained during selection of aptamers to protein HE4. The expected amplicon size is 70 bp double-stranded DNA. **M** = low molecular weight DNA ladder (ref. N3233S, New England Biolabs), **-** = negative PCR control (non template control), **+** = positive PCR control (70-mer ssDNA library as template) and samples from Cycle 1 to Cycle 10 (70-mer ssDNA aptamers as template) after ddPCR/PCR amplification and prior purification from the primers.

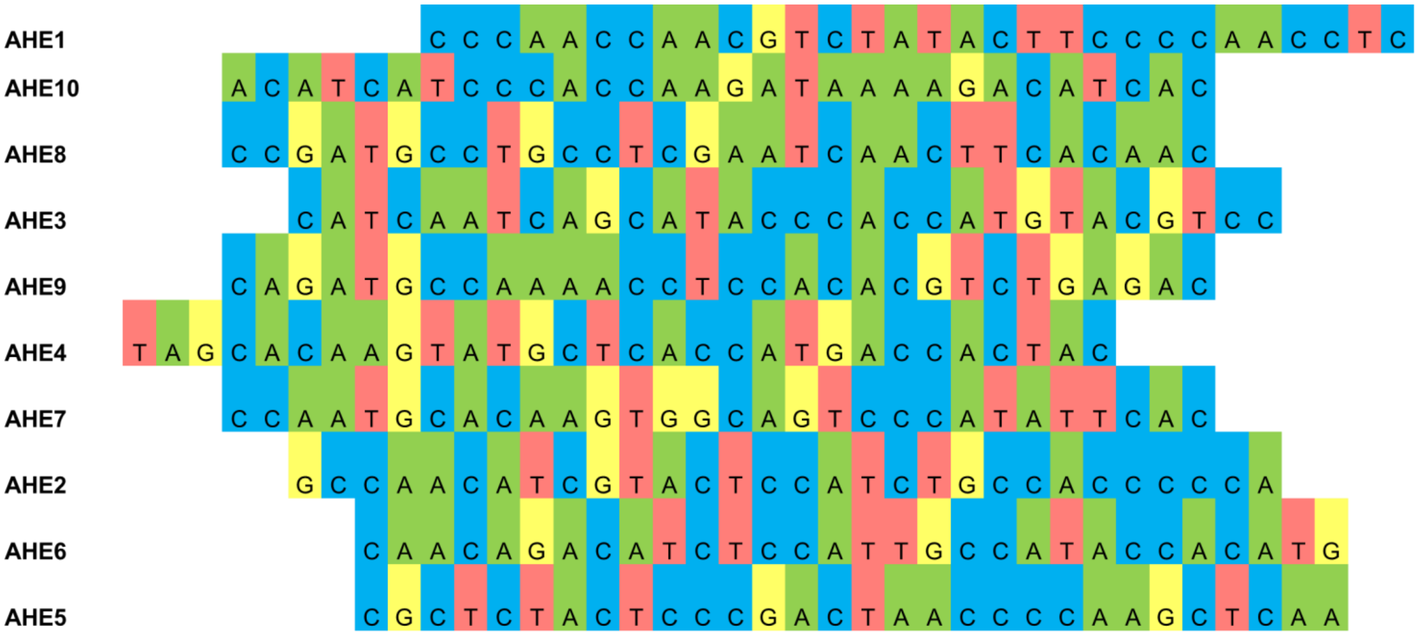

Figure S3. A. Matrix alignment of the top 10 most enriched anti-HE4 aptamer sequences. The figure present the matrix alignment of top 10 most enriched sequences (random regions) to ovarian cancer biomarker HE4. The matrix alignment did not reveal any specific shared motifs between aptamer sequences**.**

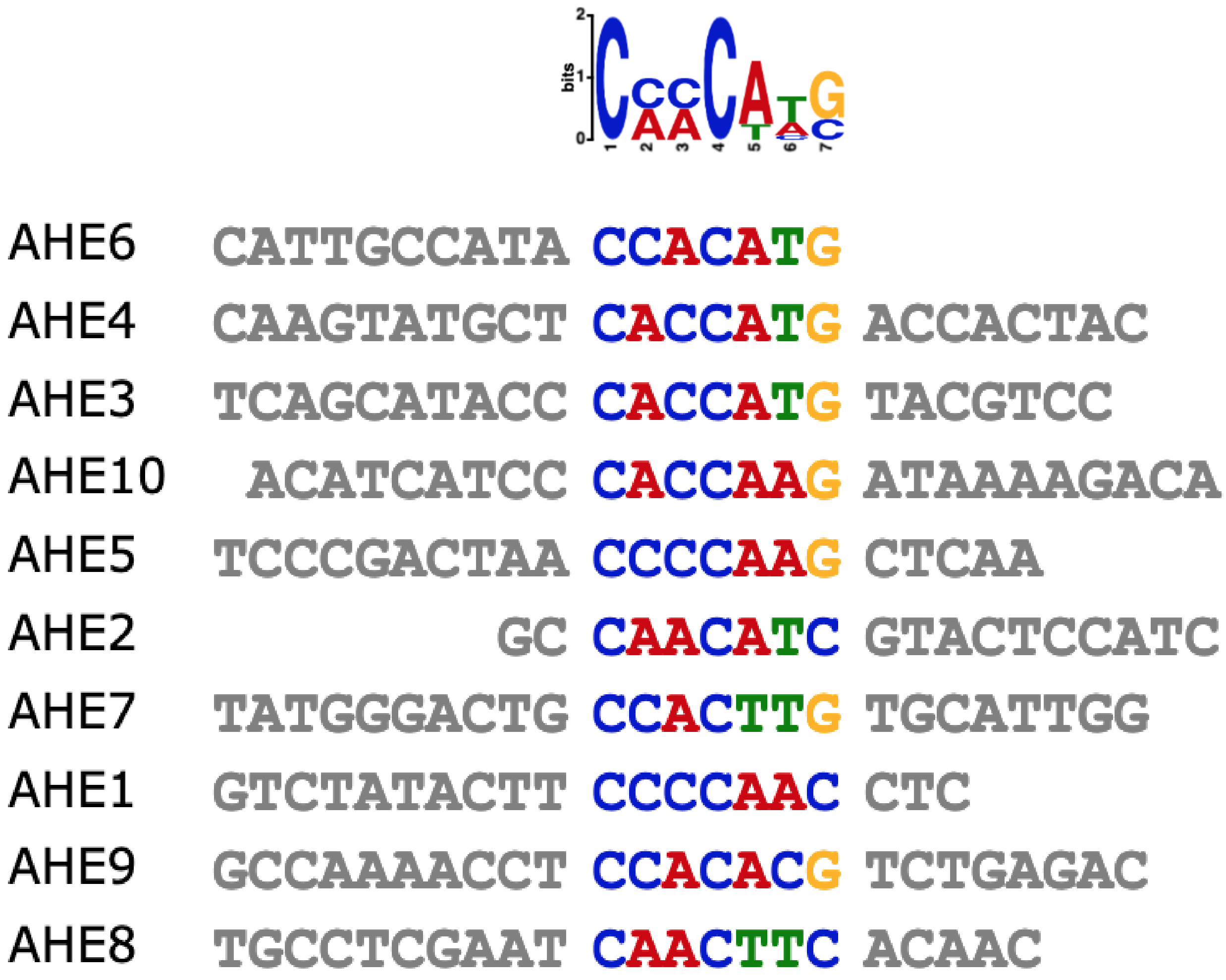

Figure S4. Conserved motif between the 10 most enriched anti-HE4 aptamer families. A conserved nucleotide motif in the 10 most enriched families was identified using the MEME suite algorithm.

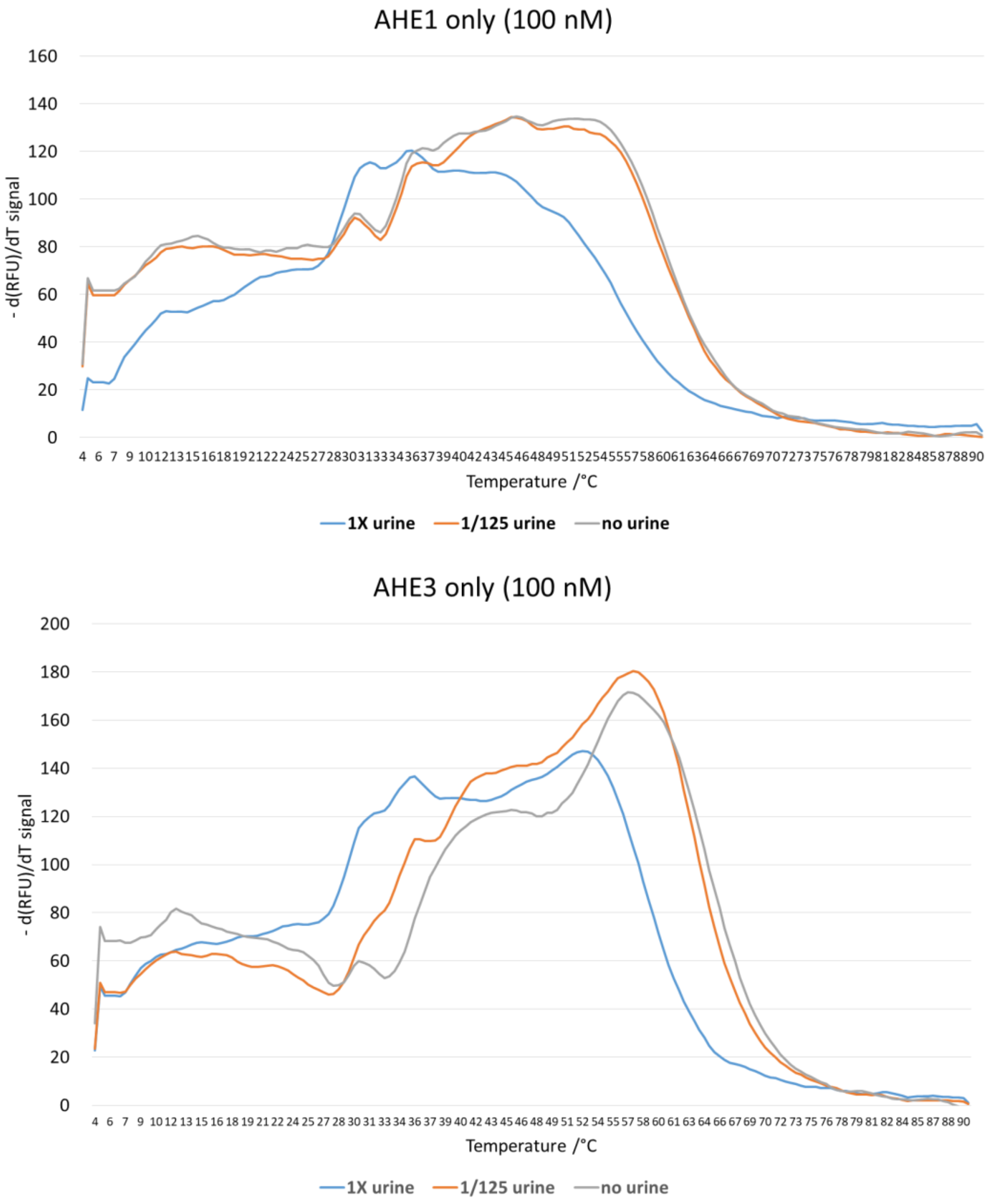

Figure S5. Effect of urine on the aptamer thermofluorimetric signal. The aptamers AHE1 and AHE3 are tested at constant concentration of 100 nM probe in 3 different conditions: undiluted urine 1X, diluted urine 1/125 and no urine (only protein buffer present). It has been shown that undiluted 1X urine decrease the −d(RFU)/dT signal in all three sequences, shifting the aptamers to lower values of T_m (potential lower stability state), suggesting interferences of concentrated urine to the analysis. Between diluted urine of 1/125 and no urine, no difference is observed. The data presented is average from four independent replicates. Therefore, all the experiments of aptamer-protein binding are performed in the presence of urine, in diluted form 1/125.

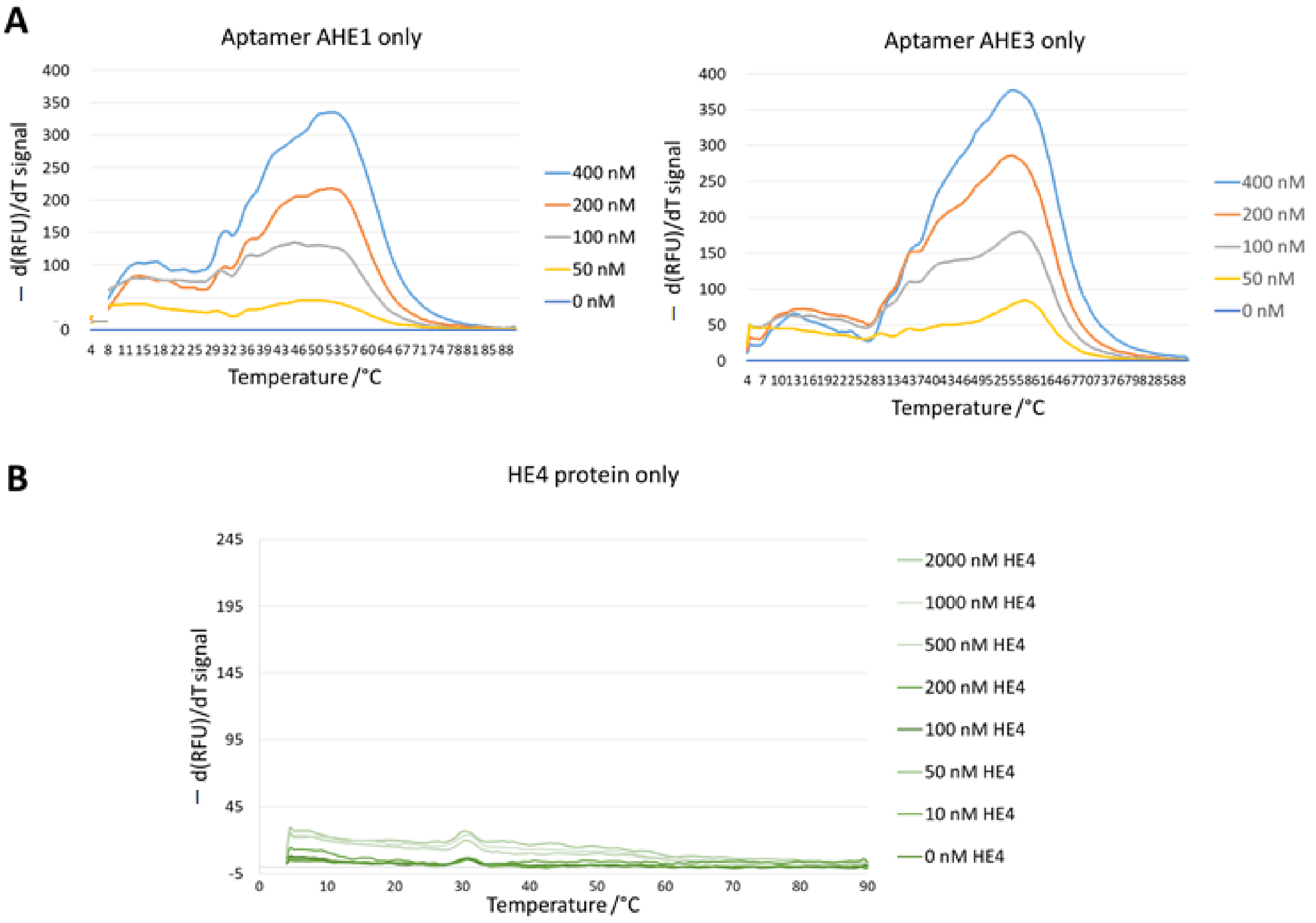

Figure S6. Control experiments of candidate aptamers and HE4 protein only prior anti-HE4 aptamers-HE4 protein binding characterization by the TFA in urine. **A. aptamers only:** The Sybr Gold is binding to the DNA aptamers with negative derivative fluorescence signal −d(RFU)/dT proportional to the aptamer quantity for AHE1 and AHE3 aptamer. The aptamers at concentration 100 nM exhibit sufficient signal intensity. The data presented is an average from four independent replicates. **B. HE4 protein only**: The Sybr Gold is not binding to the HE4 protein, the signal is negligible (just background present). The data presented is average from four independent replicates. Therefore, signal attribution to aptamers is confirmed and TFA can be used for aptamer-HE4 protein binding characterization.
